# Renpenning syndrome caused by the c.459_462delAGAG mutation in PQBP1: a case report and literature review

**DOI:** 10.3389/fgene.2026.1642438

**Published:** 2026-03-30

**Authors:** Mengting Zhang, Mengli Liu, Rongrong Wang, Fuxiang Ma, Guoshun Mao

**Affiliations:** Department of Pediatrics, Fuyang People’s Hospital of Anhui Medical University, Fuyang, Anhui, China

**Keywords:** case report, PQBP1, renpenning syndrome, whole exome sequencing, X-linked intellectual disability

## Abstract

**Background:**

Renpenning syndrome (OMIM: 309500) is a rare X-linked intellectual disability caused by variations in the polyglutamine-binding protein 1 (PQBP1) gene, characterized by moderate to severe intellectual disability, microcephaly, short stature, lean body, small testes, and abnormal facial features.

**Methods:**

Comprehensive clinical evaluation and whole exome sequencing were performed to identify the genetic basis of the clinical presentation in a 4-year-7-month-old male proband from a Chinese family. Detected variants underwent validation and familial segregation analysis by Sanger sequencing. Additionally, a literature review was conducted to analyze PQBP1-related genotype-phenotype correlations.

**Results:**

The proband exhibited typical manifestations of Renpenning syndrome, including severe global developmental delay, microcephaly, short stature, and characteristic facial features. Additionally, he presented with rare anal atresia and co-occurring autism spectrum disorder (ASD). Whole exome sequencing identified a hemizygous PQBP1 frameshift variant, NM_001032382.2:c.459_462delAGAG (p.Arg153fs) (VCV000010980.79), in the proband. Sanger sequencing confirmed this variant was maternally inherited.

**Conclusion:**

This report describes the first Chinese case of Renpenning syndrome caused by the PQBP1 c.459_462delAGAG variant, presenting with the core phenotype plus anal atresia and ASD. This case expands recognition of the clinical spectrum associated with PQBP1 variants.

## 1 Introduction

Intellectual disability (ID) is a significant public health issue that profoundly impacts society and families, with a global prevalence estimated at approximately 1%–3% (Gécz et al., 2009; Basel-Vanagaite, 2008). Notably, the X chromosome constitutes only 4% of the human genome. However, its genes are associated with about 10%–15% of genetic cases of ID (Ro and pers, 2008; Ropers and Hamel, 2005). Furthermore, about 10%–12% of male ID cases are attributed to pathogenic mutations on the X chromosome (De Luca et al., 2020; Ropers, 2010). Renpenning syndrome, caused by mutations in the polyglutamine-binding protein 1 (PQBP1) gene, represents one of the extremely rare genetic forms of X-linked intellectual disability (XLID). This syndrome was initially identified through intensive research into specific genetic pedigrees. In 1962, Renpenning’s team reported a large family spanning three generations, comprising 20 male patients who exhibited moderate to severe ID, short stature, microcephaly, dysmorphic facial features, and small testes, with no abnormalities observed in female family members (Renpenning et al., 1962). This groundbreaking study established the first direct link between ID and sex. In 2003, Kalscheuer et al. identified PQBP1 mutations in 5 out of 29 XLID families, thereby confirming the pathogenicity of this gene (Kalscheuer et al., 2003). Subsequently, PQBP1 mutations have been implicated in a range of related syndromes, including Golabi-Ito-Hall syndrome, Sutherton-Haan syndrome, Hamel-cerebro-palato-cardiac syndrome, Porteous syndrome, and non-syndromic intellectual disability (MRX55), as well as in three smaller families without reported phenotypes (Stevenson et al., 2005; Lubs et al., 2006). To honor the pioneering work of the Renpenning team, XLID caused by PQBP1 mutations was officially designated as Renpenning syndrome in 2005 (Stevenson et al., 2005).

Due to the rarity of Renpenning syndrome and the incomplete understanding of its pathogenic gene PQBP1, the global understanding of this disorder relies heavily on small-scale studies and case reports from predominantly Western populations. This study presents a case of PQBP1 gene mutation in a Chinese male patient and conducts a systematic review of existing literature. Through this analysis, the study highlights the potential phenotypic characteristics of this mutation in Asian populations, with the aim of enhancing clinicians’ awareness of the disease and providing valuable insights for diagnostic considerations.

## 2 Study subjects and methods

### 2.1 Study subjects

**FIGURE 1:**
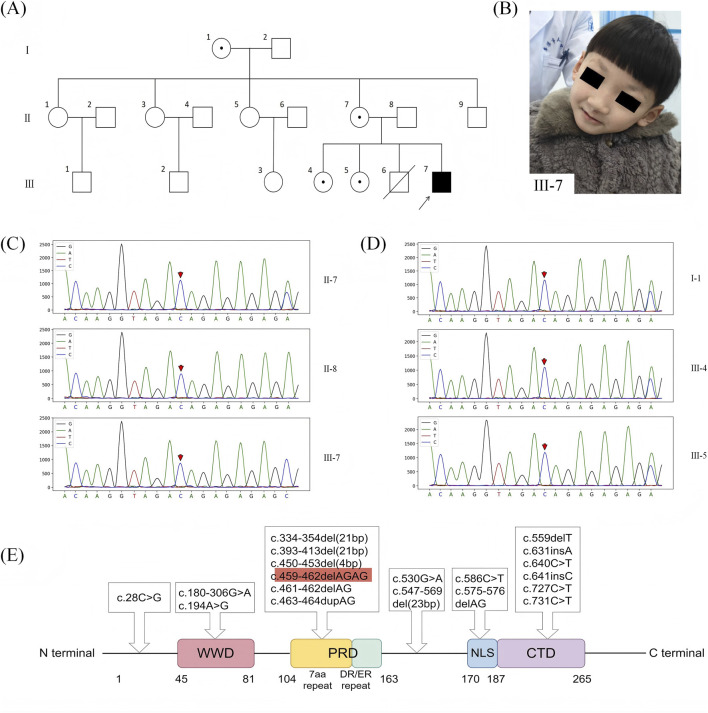
**(A)** Family pedigree: Affected males are indicated by black squares, whereas the dots indicate obligate carriers. The deceased is indicated by a slash. The arrow signifies the proband. **(B)** The facial image shows the patient with triangular face, prominent large ears, epicanthal folds, sparse eyebrows, broad nasal bridge with bulbous tip, thin upper lip, and characteristic nasal speech. **(C)** Polyglutamine-binding protein 1 (PQBP1) mutation in our case: Father (II-8): no variation in PQBP1 gene. The proband (III-7): PQBP1 c.459_462delAGAG. Mother (II-7): heterozygous. **(D)** Polyglutamine-binding protein 1 (PQBP1) mutation in our case: The maternal grandmother (I-1) and sisters (III-4 and III-5): heterozygous. **(E)** Representation of domain structure and mutations in polyglutamine-binding protein 1 (PQBP1). The detected mutation c.459_462delAGAG is shown in the red frame. 22 PQBP1 mutations were identified in previous cases, 3 mutations were not marked in the domain due to the absence of detailed data: 4.7 Mb duplication of PQBP1 at Xp11.22–p11.23; *de novo* 5 Mb duplication of PQBP1 at Xp11.22-p11.23; Xp11.2-q12. WWD, WW domain; PRD, polar amino acid-rich domain; CTD, c-terminal domain; NLS, nuclear localization signal (Figure 1E is reproduced from original illustration created by Mengting Zhang on FigDraw, with permission under copyright code TUWYR13692, by figdraw.com).

This study included a 4-year-7-month-old boy (the proband) who was admitted to the Child Growth and Health care Clinic of Fuyang People’s Hospital due to developmental delay in November 2024. The G5P4 patient was born by cesarean section at 40 weeks of gestational age with a birth weight of 3 kg and a birth length of 45 cm. Prenatal ultrasound revealed an occipitofrontal circumference (OFC) that was below the expected range for gestational age. There was no history of asphyxia or pathological jaundice at birth. Additionally, there was no history of intrauterine infection, perinatal hypoxia and asphyxia or drug exposure during pregnancy. His able-bodied, non-consanguineous parents had four children: two boys and two girls (Figure 1A). His two elder sisters were of normal intelligence, while his elder brother suffered from ID and died prematurely.

### 2.2 Methods

#### 2.2.1 Clinical data analysis

The clinical data of the proband were collected, general physical examination and specialist examination were performed, and the examination results of other hospitals and our hospital were collected, including a variety of assessment scales, genetic testing, imaging examination (brain CT, brain MRI, electroencephalogram, urinary tract color Doppler ultrasound), visual acuity and hearing test data.

#### 2.2.2 Whole exome sequencing (WES) and sanger sequencing verification

Peripheral blood was collected from the child in EDTA anticoagulant tubes. Genomic DNA was extracted using the Vazyme DNA extraction kit (#VNP-32P). DNA concentration was determined with the Yisheng Bio-dsDNA HS Assay Kit (batch 12642ES76). Sequencing libraries were constructed using the Vazyme Universal Plus DNA Library Prep Kit for MGI (V2). Target regions were captured using the EGI-Taikang Ai full-exome V3 capture kit, followed by paired-end sequencing on a BGI T7 sequencer to generate FASTQ data. Alignment was performed against the human reference genome (hg19). Data filtering used fastp, and sequence alignment used BWA. Variant database correction employed dbSNP (v159). Sequencing depth and coverage were assessed using samtools depth and GATK DepthOfCoverage. Quality control required an average depth ≥100X and ≥95% coverage at 20X. Variant calling was performed with GATK (v3.8), and GC content analysis was performed with mosdepth (v0.2.5). Variant annotation utilized ANNOVAR, integrating HGMD, HPO, and internal databases. Pathogenicity classification followed ACMG guidelines. Peripheral blood samples from the patient’s parents, siblings, and maternal grandmother underwent Sanger sequencing. Primers were designed using Primer-BLAST, amplified by PCR, purified via electrophoresis, and sequenced bidirectionally on an ABI sequencer. Sequence traces were analyzed with Chromas software.

#### 2.2.3 Literature review

A systematic search of PubMed, Web of Science, and Cochrane Library was conducted to identify relevant reports published from inception through February 2025. Search terms included “Renpenning syndrome,” “PQBP1,” “Golabi-Ito-Hall syndrome,” “Sutherland-Haan syndrome,” “Hamel cerebro-palato-cardiac syndrome,” “Porteous syndrome,” and “MRX55.” References of the included articles were also manually searched for potential studies. Only case report-related literature was included, excluding (1) reviews, mechanistic studies, other studies not related to clinical cases, and literature for which full text was not available, and (2) non-English language literature.

## 3 Results

### 3.1 Clinical data analysis

The boy was evaluated in November 2024 for speech delay and global developmental concerns. On physical examination, the child was conscious, energetic, and appeared moderately nourished. Objective measurements revealed microcephaly (OFC 46.5 cm, < −3 SD), short stature (height 99.8 cm, < −2 SD), and lean body (weight 13 kg, < −3 SD). The special examination found that the child could only speak short sentences, had poor initiative social ability, less eye contact, liked to play alone, lacked joint attention, and could not participate in group games. He also showed marked difficulties in cognitive comprehension, especially with number and color recognition, despite being able to follow simple instructions. Moreover, he often engaged in self-talk and repetitive behaviors such as spinning in circles. There were no obvious abnormalities in muscle strength and muscle tension of the limbs. He has the characteristics of a triangular face, prominent and large ears, wide inner canthus, epicanthus, semi-depilated eyebrow, bulbous nose, broad nasal bridge, thin upper lip, and nasal speech (Figure 1B). The patient has clinical diagnoses of global developmental delay (based on the Gesell assessment), microcephaly (OFC < −3 SD), short stature (height < −2 SD), and autism spectrum disorder (ASD, based on DSM-5 criteria). He regularly receives rehabilitation, including weekly speech therapy.

### 3.2 Results of clinical assessment

The patient’s significant developmental and behavioral impairments were evident across standardized assessments. Performance was in the severe range as evidenced by a Childhood Autism Rating Scale (CARS) score of 35 (moderate autism), an Autism Behavior Checklist (ABC) score of 88, and a standardized score of 5 on the Infant-Junior Middle School Student Activities of Daily Living (ADL) Scale, the latter indicating extremely severe impairment in daily functioning. Cognitive evaluations revealed significantly below-average scores. On the Wechsler Intelligence Scale for Children-Second Edition (WISC-II), the patient achieved a full-scale IQ of 52, with verbal and performance scores of 56 and 55, respectively. The Gesell Developmental Assessment (GDA) yielded a total score of 35, with subdomain scores as follows: language (29), adaptive behavior (43), gross motor (62), fine motor (53), and social competence (55). The Chinese Psychoeducational Profile-Third Edition (CPEP-3) revealed severe developmental delays in cognition (standard score 25), expressive language (17), and personal self-care (28). Moderate delays were noted across receptive language, motor skills, affective expression, social interaction, and adaptive behavior, while visual-motor imitation showed only a mild delay. Composite scores confirmed a severe delay in communication (total score 22, developmental age 20.7 months), with moderate delays in both motor (total score 33, developmental age 35.7 months) and social behavior (total score 38) domains. Additionally, the Imaginative Play Assessment (IPA) score of 4 corresponded to developmental functioning below 12 months of age.

### 3.3 Imaging and other examination results

Brain CT and MRI revealed no structural abnormalities. Electroencephalogram showed normal cortical activity, and urinary tract Doppler ultrasonography demonstrated unremarkable findings. Audiometric testing indicated normal hearing thresholds. Ophthalmologic evaluation identified bilateral refractive errors: hypermetropia (right eye: +2.75 D, left eye: +3.50 D) with compound astigmatism (right eye: −3.50 D, left eye: −0.25 D). Standard karyotyping confirmed a 46, XY chromosomal constitution. Chromosomal microarray analysis (CMA) detected no clinically significant copy number variations (CNVs).

### 3.4 Results of WES

WES showed that the proband carried a hemizygous frameshift mutation of PQBP1 gene NM_001032382.2:c.459_462delAGAG (p.Arg153fs) (VCV000010980.79). Sanger sequencing confirmed that the hemizygous variant was inherited from his mother (heterozygous carrier), but not from his father (Figure 1C). The variant was rated as pathogenic according to ACMG guidelines (PVS1+PS3+PS4+PM2_Supporting + PP1). No CNV or chromosomal abnormalities related to the patient’s clinical phenotype were detected.

### 3.5 Family co-segregation verification

Sanger sequencing showed that the maternal grandmother and two elder sisters of the proband carried a heterozygous mutation of PQBP1 c.459_462delAGAG (Figure 1D), which was consistent with the X-linked recessive inheritance pattern.

### 3.6 Literature review

The human PQBP1 gene is located at Xp11.23 and encodes a 265-amino acid protein consisting of a WW domain (WWD), a polar amino acid-rich domain (PRD), and a C-terminal domain (CTD) with a nuclear localization signal (NLS) (Cheng et al., 2023). Summarizing the previously published literature, 22 mutations have been found in PQBP1 (Renpenning et al., 1962; Kalscheuer et al., 2003; Stevenson et al., 2005; Lubs et al., 2006; Deqaqi et al., 1998; Lenski et al., 2004; Bonnet et al., 2006; Cossée et al., 2006; Fichera et al., 2005; Martínez-Garay et al., 2006; Sheen et al., 2010; Germanaud et al., 2011; Rejeb et al., 2011; Flynn et al., 2010; Redin et al., 2014; Abdel-Salam et al., 2018; Jeo et al., 2018; Mameesh et al., 2019; Cho et al., 2020; Masih et al., 2019; Kurt et al., 2020; Aleksic et al., 2021; Murdock et al., 2021; Lopez-Martín et al., 2022; Kaymakçalan et al., 2022; Kanagavel and Manokaran, 2025; Pan et al., 2025). These mutations can be divided into five groups based on the domains affected: Missense mutations in the WWD, gross deletions in the PRD, AG nucleotide duplications or deletions affecting DR/ER repeats in the PRD, AG nucleotide deletions and nonsense mutations affecting the NLS, and insertion and missense mutations in the CTD. Additional details of included studies are shown in Table 1; Figure 1E.

**TABLE 1:** PQBP1 mutation sites and clinical features in patients with Renpenning syndrome.

References	Syndrome/family	ClinVar ID	Mutation	Domain	Clinical features
Renpenning et al. (1962)	original family	VCV004795965.1	c.641insC^*^	C-terminal region	ID, microcephaly, long face, short stature, lean body, small testes
Kalscheuer et al. (2003), Deqaqi et al. (1998)	MRX55	VCV000010980.79	c.459_462delAGAG	DR/ER repeat	ID, short stature
Kalscheuer et al. (2003)	N45	VCV000010980.79	c.459_462delAGAG	DR/ER repeat	ID, microcephaly, short stature, lean body, dysmorphic facies, anal atresia, complete situs inversus
Kalscheuer et al. (2003)	Hamel cerebro-palato-cardiac (N40)	VCV000010981.7	c.461_462delAG	DR/ER repeat	ID, microcephaly, short stature, long face, lean body, congenital heart defect
Kalscheuer et al. (2003)	Sutherland_Haan syndrome	VCV000010979.11	c.463_464dupAG^*^	DR/ER repeat	ID, microcephaly, short stature, long face, lean body, small testes, anal stenosis or atresia
Kalscheuer et al. (2003)	N09	VCV000010979.11	c.463_464dupAG^*^	DR/ER repeat	ID, microcephaly, long face, lean body
Lenski et al. (2004)	K9008	VCV001691229.3	c.575_576delAG	NLS	ID, microcephaly, short stature, long face, lean body
Stevenson et al. (2005)	K8600	VCV000010980.79	c.459_462delAGAG	DR/ER repeat	ID, microcephaly, lean body, dysmorphic facies, small testes, developmental delay
Stevenson et al. (2005)	Porteous syndrome	VCV000010979.11	c.463_464dupAG^*^	DR/ER repeat	ID, long face, lean body
Lubs et al. (2006)	Golabi_Ito_Hall (K8275 family)	VCV000010985.4	c.194A>G	WWD	ID, microcephaly, lean body, short stature, postnatal growth deficiency, atrial septal defect, spastic diplegia, triangular face
Cossée et al. (2006)	F1	VCV000010983.1	c.547_569del	After the DR/ER repeat	ID, microcephaly, lean body, dysmorphic facies, bilateral choanal atresia, and anal atresia
Cossée et al. (2006)	F2, F3 and F4	VCV000010984.32	c.334_354del	PRD	ID, behavioral anomalies
Cossée et al. (2006)	F5	VCV000284597.20	c.393_413del	PRD	ID, microcephaly, lower limbs spasticity
Martínez-Garay et al. (2006)	Martinez_Garay family	VCV000010981.7	c.461_462delAG	DR/ER repeat	ID, microcephaly, short stature, microphthalmia
Sheen et al. (2010)	Sheen family	VCV000010980.79	c.459_462delAGAG	DR/ER repeat	ID, microcephaly, short stature, dysmorphic facies, hearing loss, periventricular heterotopia
Germanaud et al. (2011)	P family	VCV000010980.79	c.459_462delAGAG	DR/ER repeat	ID, microcephaly, short stature, lean body, dysmorphic facies, muscular atrophy
Germanaud et al. (2011)	CB family	VCV000010980.79	c.459_462delAGAG	DR/ER repeat	ID, microcephaly, short stature, lean body, dysmorphic facies, muscular atrophy
Germanaud et al. (2011)	S family	VCV000010980.79	c.459_462delAGAG	DR/ER repeat	ID, microcephaly, short stature, lean body, dysmorphic facies
Germanaud et al. (2011)	B family	VCV000010980.79	c.459_462delAGAG	DR/ER repeat	ID, microcephaly, short stature, lean body, dysmorphic facies, muscular atrophy
Germanaud et al. (2011)	AH family	VCV000010980.79	c.459_462delAGAG	DR/ER repeat	ID, microcephaly, short stature, lean body, dysmorphic facies
Germanaud et al. (2011)	L family	VCV000545093.14	c.586C>T	NLS	ID, microcephaly, short stature, lean body, dysmorphic facies, muscular atrophy
Rejeb et al. (2011)	Tunisian family	VCV004795964.1	c.631insA^*^	C-terminal region	ID, microcephaly, long face, short stature, lean body
Jeo et al. (2018)	Korean family	VCV004795963.1	c.559delT	NLS	ID, short stature, and microcephaly
Redin et al. (2014)	Autism liked	VCV000235848.3	c.731C>T	C-terminal region	Moderate ID, poor autonomy, communication and social interaction disorders, learning difficulties, autistic behavior
Abdel-Salam et al. (2018)	North African family	VCV001527945.4	c.530G>A	After the DR/ER repeat	the frontal and bitemporal baldness, bulbous nose, long palpebral fissures, cupped ears, pre_auricular tags, simple helix and pitted ear lobules, partial agenesis of the corpus callosum
Mameesh et al. (2019)	Omani family	VCV000284240.7	c.450_453del	DR/ER repeat	microcephaly, mild short stature, triangular face, unilateral microphthalmos, large bulbous nose, cup_shaped ears, short philtrum
Cho et al. (2020)	a female patient	VCV000010980.79	c.459_462delAGAG	DR/ER repeat	ID, microcephaly, short stature, lean body, dysmorphic facies, hearing loss, and unilateral microphthalmia with cataract
Masih et al. (2019)	an Indian patient	VCV000010980.79	c.459_462delAGAG	DR/ER repeat	microcephaly, short stature, lean body, dysmorphic facies, developmental delay, progressive atrophy of the upper back muscles, loss of cervical lordosis, and upper thoracic scoliosis
Kurt et al. (2020)	a Turkish Patient	VCV001308367.8	c.640C>T	C_terminal region	ID, microcephaly, short stature, small testes, dysmorphic facies, speech and language problems, patent foramen ovale, recurrent urinary tract infections and bronchitis, hypogammaglobulinemia
Aleksic et al. (2021)	Serbian family	VCV000010980.79	c.459_462delAGAG	DR/ER repeat	ID, microcephaly, short stature, dysmorphic facies, cardiac abnormalities, developmental delay, seizures, asymmetric cerebellar hemispheres
Murdock et al. (2021)	A male child patient	VCV000977747.2	c.180-306G>A	WWD	ID, microcephaly, short stature, lean body, dysmorphic facies, developmental delay, congenital heart defects (ventricular septal defect and patent ductus arteriosus), vertebral anomalies (butterfly vertebrae), ectopic pelvic left kidney, hypospadias, sensorineural hearing loss
Lopez-Martín et al. (2022)	Spanish family	VCV000977914.4	c.727C>T	C_terminal region	dysmorphic facies, Attention_deficit/hyperactivity disorder, borderline intellectual functioning with marked language impairment
Kaymakçalan et al. (2022)	Turkish family	VCV000010980.79	c.459_462delAGAG	DR/ER repeat	ID, microcephaly, short stature, dysmorphic facies, congenital heart disease, developmental delay
Kanagavel and Manokaran (2025)	an Indian patient	VCV000010980.79	c.459_462delAGAG	DR/ER repeat	ID, microcephaly, short stature, long face, lean body, dysmorphic facies, developmental delay
Pan et al. (2025)	Chinese family	VCV004795962.1	c.28C>G	outside the usual functional regions	ID, microcephaly, short stature, lean body, dysmorphic facies, developmental delay, speech delay, eecurrent febrile convulsions, henoch_schonlein purpura, allergic reaction, memory impairment, brain MRI scans indicated demyelination

Abbreviation: ID, intellectual disability.

* Variant nomenclature has been updated to conform to current HGVS, guidelines. In the ClinVar records: c.641insC has been revised to c.641_642insC; c.631insA has been updated to c.632dup; and c.463_464dupAG, has been revised to c.461_462dupAG.

After a detailed search, nine case reports (Kalscheuer et al., 2003; Stevenson et al., 2005; Sheen et al., 2010; Germanaud et al., 2011; Cho et al., 2020; Masih et al., 2019; Aleksic et al., 2021; Kaymakçalan et al., 2022; Kanagavel and Manokaran, 2025) of c.459_462delAGAG mutations in the PQBP1 gene were included, totaling 27 patients. All cases had moderate to severe ID except for one adult male patient who did not undergo routine intelligence testing. Additionally, developmental delay constituted the most frequent manifestation at 96%, followed sequentially by microcephaly in 89% of patients, lean body build in 70%, and short stature in 67%. Characteristic dysmorphic facial features included a long, triangular, or narrow face; large and cupped ears; a bulbous, large, or prominent nose; and sparse lateral eyebrows. The remaining details are shown in Table 2 and Table 3.

**TABLE 2:** Clinical features of Renpenning syndrome patients with PQBP1 c.459_462delAGAG mutation.

References	syndrome /Family	Ancestry	Number of cases	ID	Microce-phaly	Short stature	Lean body	Small testis	Develop-mental delay	Dysmorphic face	Anal atresia	Cardiac anomaly	Others	Mutation origin
Kalscheuer et al. (2003)	MRX55	Moroccan	3	+	-	+	-	-	+	Large narrow face, bulbous nose, short philtrum, and overhanging columella	-	-	-	N
Kalscheuer et al. (2003)	N45	Dutch	3	+	+	+	+	-	+	Long narrow face, malar hypoplasia, prognathism, large or protruding ears, high-arched palate, upslanting palpebral fissures, and nasal speech	+	-	Complete situs inversus	N
Stevenson et al. (2005)	K8600	N	4	+	+	-	+	+	+	Upslanting palpebrae, long narrow face, large cupped ears, cleft palate, central balding, and epicanthus	-	+	Autism spectrum disorder, depression, systolic and diastolic heart murmurs, hypertension and mitral valve prolapse in adulthood	N
Sheen et al. (2010)	sheen family	African American	2	+	+	+	-	-	+	Broadened nose, narrow chin, freckled tongue, low-set and cupped ears	-	-	Impaired hearing, mild cognitive impairment, chronic cough, flat feet, multiple sub-centimeter pulmonary nodules on chest CT, and nodular gray matter heterotopia along the trigones of the lateral ventricles on MR imaging	maternal
Germanaud et al. (2011)	P family	French	1	+	+	+	+	-	+	Long or triangular face, sparse lateral eyebrows, ears (large, protruding, mildly dysplastic), thin upper lip, prognathism/micro retrognathism jaw, long/curved eyelashes, and malar hypoplasia	-	-	Muscular atrophy (back), hypermetropia, strabismus, terminal spine defect, MCP-1 ankylosis, velar dysfunction	N
Germanaud et al. (2011)	CB family	French	2	+	+	+	+	+	+	Long or triangular face, sparse lateral eyebrows, ears (large, protruding, mildly dysplastic), rough and slightly sparse hair, prominent and large nose, prognathism/micro retrognathism jaw, and long/curved eyelashes	+	-	Muscular atrophy (back and hand), phimosis, lower pyramidal signs, terminal spine defect, MCP-1 ankylosis, velar dysfunction, autism spectrum disorder	N
Germanaud et al. (2011)	S family	French	2	+	+	+	+	-	+	Long or triangular face, sparse lateral eyebrows, ears (large, protruding, mildly dysplastic), rough and slightly sparse hair, prominent and large nose, thin upper lip, upslanting palpebral fissures, and malar hypoplasia	-	+	Muscular atrophy (back), pronosupination restriction, horseshoe kidney, phimosis, strabismus, lower pyramidal signs, hypermetropia, terminal spine defect, MCP-1 ankylosis, velar dysfunction	N
Germanaud et al. (2011)	B family	French	2	+	+	+	+	-	+	Long or triangular face, sparse lateral eyebrows, ears (large, protruding, mildly dysplastic), rough and slightly sparse hair, prominent and large nose, thin upper lip, prognathism/micro retrognathism jaw, upslanting palpebral fissures, and malar hypoplasia	-	-	Muscular atrophy (back and hand), lower pyramidal signs, pronosupination restriction, horseshoe kidney, hypospadias, terminal spine defect, MCP-1 ankylosis, velar dysfunction	N
Germanaud et al. (2011)	AH family	French	3	+	+	+	+	-	+	Long or triangular face, sparse lateral eyebrows, ears (large, protruding, mildly dysplastic), rough and slightly sparse hair, prominent and large nose, thin upper lip, prognathism/micro retrognathism jaw, upslanting palpebral fissures, long/curved eyelashes, and malar hypoplasia	-	-	Muscular atrophy (back), MCP-1 ankylosis, strabismus, lower pyramidal signs, hypermetropia, terminal spine defect, velar dysfunction	N
Cho et al. (2020)	a female patient	South Asian	1	+	+	+	+	female	+	Long and triangular face, upslanting palpebral fissures, prominent nasal tip, short philtrum	-	-	Unilateral ocular anomalies (right microphthalmia and cataract), bilateral mild hearing loss, linear streaks of cutaneous hypopigmentation, increased peripheral tone	*de novo*, paternal X chromo-some
Masih et al. (2019)	an Indian patient	Indian	1	N	+	+	+	-	+	Long, narrow face with deep-set eyes, hypotelorism (narrow distance between eyes), malar hypoplasia (underdeveloped cheekbones), prominent nose with a convex nasal ridge, protruding ears	+	-	Muscular atrophy of the upper back, loss of cervical lordosis, mild thoracic scoliosis with curvature toward the left, narrow anterior-posterior diameter of the thorax, prominent scapulae (winged scapulae), long and slender digits with joint prominence, constriction at the base of both fourth fingers, sandle gaps, second toes longer than the great toes, limited cognitive abilities, mild microcytic hypochromic anemia, ultrasound suggested mild increase in renal parenchymal echogenicity	maternal
Aleksic et al. (2021)	Serbian family	Serbian	1	+	+	+	-	-	+	Narrow face, midfacial and maxillary hypoplasia, short philtrum, low-set ears, low-hanging columella, high palate, and wide nasal bridge	-	+	Upper and lower extremities hypotonia with joint hypermobility, seizures, asymmetric cerebellar hemispheres, specifically mild right cerebellar hemisphere hypoplasia on brain MRI	maternal
Kaymakçalan et al. (2022)	Turkish family	Turkish	1	+	+	+	-	-	+	Long and narrow face, bulbous nose, sparse lateral eyebrows, strabismus (crossed eyes)	-	+	Tetralogy of fallot with pulmonary atresia, muscular atresia, hypoplasia of the main pulmonary artery, recurrent infective endocarditis	maternal
Kanagavel and Manokaran (2025)	an Indian patient	Indian	1	+	+	+	+	-	+	Narrow and tall face, upslanting palpebral fissures, bulbous nose tip, and cupped ears	-	-	-	maternal
Present study	a Chinese patient	Chinese	1	+	+	+	+	-	+	Triangular face, wide inner canthus, epicanthus, bulbous nose, prominent and large ears, semi-depilated eyebrow, broad nasal bridge, thin upper lip	+	-	Autism spectrum disorder	maternal

N, no data; +, positive; -, negative.

Abbreviation: ID, intellectual disability.

**TABLE 3:** Common clinical manifestations associated with PQBP1 c.459_462delAGAG mutation in Renpenning syndrome.

Manifestations		Number of cases
ID	​	26/27
microcephaly	​	24/27
short stature	​	18/27
lean body	​	19/27
small testes	​	3/27
dysmorphic facies	​	​
​	long/triangular/narrow face	22/27
​	malar hypoplasia	9/27
​	sparse lateral eyebrows	11/27
​	long or curved eyelashes	6/27
​	epicanthus	1/27
​	upslanting palpebral fissures	9/27
​	large/cupped/protruding/low-set ears	21/27
​	broadened/bulbous/large/prominent nose	15/27
​	thin upper lip	6/27
​	cleft or highly arched palate	5/27
​	prognathism/micro retrognathism jaw	10/27
​	rough and slightly sparse hair	10/27
anal atresia	​	3/27
cardiac anomaly	​	4/27
muscular atrophy	​	10/27
developmental delay	​	26/27

Abbreviation: ID, intellectual disability.

## 4 Discussion

This is the first case report of PQBP1 gene c.459_462delAGAG mutation in a Chinese family with Renpenning syndrome. The typical clinical features of Renpenning syndrome are ID, microcephaly, short stature, lean body, and small testes type (Renpenning et al., 1962). Reviewing the previous cases of the same type of gene locus, in addition to the typical clinical features, patients with this gene locus mutation can present with muscle atrophy, developmental delay, hearing and vision abnormalities, cardiac abnormalities, situs inversus, etc (Kalscheuer et al., 2003; Germanaud et al., 2011; Kanagavel and Manokaran, 2025). Similar clinical features were observed in our patient, who presented with global developmental delay, microcephaly, short stature, lean body, dysmorphic facies, anal atresia, and ASD. The patient’s maternal grandmother, mother, and two siblings were all carriers but had no obvious clinical manifestations. A pathogenic hemizygous deletion in exon 5 of the PQBP1 gene (c.459_462delAGAG p. R153fs*41) was identified by clinical WES, resulting in premature protein termination of 41 amino acids downstream of codon 153. The male hemizygous phenotype is exomorphic, and the female carriers do not show typical symptoms due to skewed X chromosome inactivation, which is consistent with literature reports (Lopez-Martín et al., 2022). It is worth noting that this gene mutation site has been reported in a female case (Cho et al., 2020), whose clinical manifestations were consistent with the typical manifestations of Renpenning syndrome. The combined results of the following factors were considered: (1) *de novo* mutations; (2) X chromosome inactivation (XCI) tilt; (3) variable XCI mode; (4) expression of mutant transcripts.

Different mutations affect different domains and lead to different clinical symptoms. The WWD with a wide array of functions recognizes proline-rich proteins, including the splicing factor WBP11 (Pucheta-Martinez et al., 2016). The WWD directs involvement in several XLID, including Golabi-Ito-Hall syndrome, where a single point mutation (Y65C) correlates with the development of the disease. The Y65C missense mutation was the first identified in PQBP1 (Lubs et al., 2006). This mutation damages the folding of the WWD and its protein binding ability, which results in compromised binding with WBP11 and reduced splicing efficiency (Sudol et al., 2012; Tapia et al., 2010). Patients with the Y65C mutation show severe ID, microcephaly, postnatal growth deficiency, and atrial septal defect. The deletion of multiple nucleotides in the PRD and NLS and the insertion of the repeat AG in the PRD affecting the dinucleotide of the DR/ER repeat resulted in a frameshift, leading to premature stop codons and the production of truncated proteins. These mutations mainly cause the symptoms of Renpenning syndrome, including microcephaly, mild-to-severe intellectual disability, short stature, emaciation, and small testes. In addition, deletion of the AG nucleotide in PRD can lead to unilateral microphthalmia (Mameesh et al., 2019; Cho et al., 2020). Mutations in the CTD, such as insertions and missense mutations, disrupt protein interactions, leading to altered splicing activity. Patients with CTD mutations often present with symptoms consistent with Renpenning syndrome and can also present with attention deficit-hyperactivity disorder, ASD, and language deficits (Redin et al., 2014; Lopez-Martín et al., 2022).

PQBP1 is a highly conserved protein associated with neurodegenerative disorders (Wang et al., 2013). Mutations in the human PQBP1 gene have been shown to cause ID, microcephaly, and other symptoms (Kalscheuer et al., 2003; Germanaud et al., 2011). Our patient was associated with ASD in addition to typical symptoms, similar to the clinical presentation of the patient in the case report by (Stevenson et al., 2005; Růzicka et al., 2005). Targeted high-throughput sequencing identified a PQBP1-P244L missense mutation (c.731C>T, P244L) in ID patients (Redin et al., 2014). These patients present with moderate ID, poor autonomy, communication and social interaction impairments, learning difficulties, and overt autistic behaviors. Liu et al. (Liu et al., 2020) showed that the PQBP1-P244L mutant disrupted the interaction with TXNL4A. This may explain the presence of ASD in patients with PQBP1 mutations. Nevertheless, no large sample studies have definitively supported PQBP1 as a core pathogenic gene for ASD. Autistic symptoms may be secondary to severe cognitive impairment or comorbid phenomena rather than being directly causal. Manifestations such as anal atresia and hyperopia are uncommon in patients with Renpenning syndrome, and the association with PQBP1 mutations still needs to be further explored.

Renpenning syndrome follows an X-linked recessive inheritance pattern. If a mutation is identified, prenatal diagnosis should be recommended for parents in further pregnancies. Genetic tests such as WES and other methods of genetic analysis can identify female carriers and inform them of the risk of transmitting the gene to their offspring. Such tests can guide affected families on the importance of reproduction, especially the possibility of having healthy offspring through assisted reproductive technologies and prenatal diagnostics. Although there is currently no cure for Renpenning syndrome, early education and intervention can be provided by trained therapists. Treatment of related symptoms and malformations, such as heart defects, hypospadias, conductive hearing loss, anal atresia, strabismus, etc., can greatly improve the patient’s life and minimize mortality. In addition, children are required to attend special education classes in school due to learning, cognitive and other impairments. Ito et al. (Ito et al., 2009) suggested that administration of 4-phenylbutyric acid (PBA), an HDAC inhibitor, efficiently improved the expression of these genes and rescued the abnormal phenotypes in adult PQBP1-knockdown mice. This finding was unexpected and indicated that PQBP1 dysfunction in regulating gene expression may underlie the abnormal behavior and cognition of PQBP1-knockdown mice and that recovery of expression of such PQBP1 target genes might improve the symptoms in adult patients with Renpenning syndrome and PQBP1-linked ID.

In conclusion, this study described the case of Renenning syndrome from a Chinese family with the c.459_462delAGAG mutation in PQBP1. Patients with ID and developmental disabilities should be alerted, and systematic identification of the disease could facilitate early clinical intervention. The mechanisms and clinical manifestations of different PQBP1 mutations remain to be further investigated.

## Data Availability

The original contributions presented in the study are included in the article/Supplementary Material, further inquiries can be directed to the corresponding author.

## References

[B1] Abdel-Salam G. M. H. Miyake N. Abdel-Hamid M. S. Sayed I. S. M. Gadelhak M. I. Ismail S. I. (2018). Phenotypic and molecular insights into PQBP1‐related intellectual disability. Am. J. Med. Genet. A 176, 2446–2450. 10.1002/ajmg.a.40479 30244542

[B2] Aleksic D. Borkovic M. Krivacic J. Petrusic I. Milic Rasic V. (2021). Frameshift mutation in polar Rich Domain (PRD) of PQBP1 gene associated with asymmetric cerebellar hemispheres: a case report of renpenning syndrome. Inn. J. Pediatr. 31, e111431. 10.5812/ijp.111431

[B3] Basel-Vanagaite L. (2008). Clinical approaches to genetic mental retardation. Isr. Med. Assoc. J. 10, 821–826. 19070297

[B4] Bonnet C. Grégoire M. J. Brochet K. Raffo E. Leheup B. Jonveaux P. (2006). Pure *de-novo* 5 Mb duplication at Xp11.22–p11.23 in a male: phenotypic and molecular characterization. J. Hum. Genet. 51, 815–821. 10.1007/s10038-006-0023-3 16900295

[B5] Cheng S. Liu X. Yuan L. Wang N. Zhang Z. C. Han J. (2023). The role of PQBP1 in neural development and function. Biochem. Soc. Trans. 51, 363–372. 10.1042/BST20220920 36815699

[B6] Cho R. Y. Peñaherrera M. S. Du Souich C. Huang L. Mwenifumbo J. Nelson T. N. (2020). Renpenning syndrome in a female. Am. J. Med. Genet. A 182, 498–503. 10.1002/ajmg.a.61451 31840929

[B7] Cossée M. Demeer B. Blanchet P. Echenne B. Singh D. Hagens O. (2006). Exonic microdeletions in the X-linked PQBP1 gene in mentally retarded patients: a pathogenic mutation and in-frame deletions of uncertain effect. Eur. J. Hum. Genet. 14, 418–425. 10.1038/sj.ejhg.5201593 16493439

[B8] De Luca C. Race V. Keldermans L. Bauters M. Van Esch H. (2020). Challenges in molecular diagnosis of X-linked intellectual disability. Br. Med. Bull. 133, 36–48. 10.1093/bmb/ldz039 32043524

[B9] Deqaqi S. C. N'Guessan M. Forner J. Sbiti A. Beldjord C. Chelly J. (1998). A gene for non-specific X-linked mental retardation (MRX55) is located in Xp11. Ann. Genet. 41, 11–16. 9599645

[B10] Fichera M. Falco M. Lo G. M. Castiglia L. Guarnaccia V. Calì F. (2005). Skewed x‐inactivation in a family with mental retardation and PQBP1 gene mutation. Clin. Genet. 67, 446–447. 10.1111/j.1399-0004.2005.00436.x 15811016

[B11] Flynn M. Zou Y. S. Milunsky A. (2010). Whole gene duplication of the PQBP1 gene in syndrome resembling renpenning. Am. J. Med. Genet. A 155, 141–144. 10.1002/ajmg.a.33756 21204222

[B12] Gécz J. Shoubridge C. Corbett M. (2009). The genetic landscape of intellectual disability arising from chromosome X. Trends Genet. 25, 308–316. 10.1016/j.tig.2009.05.002 19556021

[B13] Germanaud D. Rossi M. Bussy G. Gérard D. Hertz-Pannier L. Blanchet P. (2011). The renpenning syndrome spectrum: new clinical insights supported by 13 new PQBP1-mutated males. Clin. Genet. 79, 225–235. 10.1111/j.1399-0004.2010.01551.x 20950397

[B14] Ito H. Yoshimura N. Kurosawa M. Ishii S. Nukina N. Okazawa H. (2009). Knock-down of PQBP1 impairs anxiety-related cognition in mouse. Hum. Mol. Genet. 18, 4239–4254. 10.1093/hmg/ddp378 19661183

[B15] Jeong H. I. Yang A. Kim J. Jang J. H. Cho S. Y. Jin D. K. (2018). First Korean case of renpenning syndrome with novel mutation in PQBP1 diagnosed by targeted exome sequencing, and literature review. Ann. Clin. Lab. Sci. 48, 522–527. 30143497

[B16] Kalscheuer V. M. Freude K. Musante L. Jensen L. R. Yntema H. G. Gécz J. (2003). Mutations in the polyglutamine binding protein 1 gene cause X-linked intellectual disability. Nat. Genet. 35, 313–315. 10.1038/ng1264 14634649

[B17] Kanagavel Y. Manokaran R. K. (2025). Distinct facial dysmorphisms in a child with renpenning syndrome. Ann. Indian Acad. Neurol. 28, 110–111. 10.4103/aian.aian_789_24 39779256 PMC11892980

[B18] Kaymakçalan H. Ercan-Şençiçek A. G. Cebeci A. N. Dong W. Yalım Yalçın A. S. (2022). A rare etiology of tetralogy of Fallot with pulmonary atresia: renpenning syndrome. Anatol. J. Cardiol. 26, 149–150. 10.5152/AnatolJCardiol.2021.554 35190366 PMC8878915

[B19] Kurt C. F. Eyerci N. Aytekin C. Eksioglu A. S. (2020). Renpenning Syndrome in a Turkish Patient: *de novo* Variant c.607C>T in PACS1 and Hypogammaglobulinemia Phenotype. Mol. Syndromol. 11, 157–161. 10.1159/000507562 32903913 PMC7445573

[B20] Lenski C. Abidi F. Meindl A. Gibson A. Platzer M. Frank Kooy R. (2004). Novel truncating mutations in the polyglutamine tract binding protein 1 gene (PQBP1) cause renpenning syndrome and X-Linked mental retardation in another family with Microcephaly. Am. J. Hum. Genet. 74, 777–780. 10.1086/383205 15024694 PMC1181956

[B21] Liu X. Dou L. X. Han J. Zhang Z. C. (2020). The renpenning syndrome-associated protein PQBP1 facilitates the nuclear import of splicing factor TXNL4A through the karyopherin β2 receptor. J. Biol. Chem. 295, 4093–4100. 10.1074/jbc.RA119.012214 32041777 PMC7105315

[B22] Lopez-Martín S. Albert J. Peña Vila-Belda M. D. M. Liu X. Zhang Z. C. Han J. (2022). A mild clinical and neuropsychological phenotype of renpenning syndrome: a new case report with a maternally inherited PQBP1 missense mutation. Appl. Neuropsychol. Child. 11, 921–927. 10.1080/21622965.2021.1970551 34470565

[B23] Lubs H. Abidi F. E. Echeverri R. Holloway L. Meindl A. Stevenson R. E. (2006). Golabi-Ito-Hall syndrome results from a missense mutation in the WW domain of the PQBP1 gene. J. Med. Genet. 43, e30. 10.1136/jmg.2005.037556 16740914 PMC2564547

[B24] Mameesh M. M. Al-Kindy A. Al-Yahyai M. Ganesh A. (2019). Microphthalmos-anophthalmos-coloboma (MAC) spectrum in two brothers with renpenning syndrome due to a truncating mutation in the polyglutamine tract binding protein 1 (PQBP1) gene. Ophthalmic Genet. 40, 534–540. 10.1080/13816810.2019.1686158 31718390

[B25] Martínez-Garay I. Tomás M. Oltra S. Ramser J. Moltó M. D. Prieto F. (2006). A two base pair deletion in the PQBP1 gene is associated with microphthalmia, microcephaly, and mental retardation. Eur. J. Hum. Genet. 15, 29–34. 10.1038/sj.ejhg.5201717 17033686

[B26] Masih S. Moirangthem A. Phadke S. R. (2019). Renpenning syndrome in an Indian patient. Am. J. Med. Genet. A 182, 293–295. 10.1002/ajmg.a.61457 31840915

[B27] Murdock D. R. Dai H. Burrage L. C. Rosenfeld J. A. Ketkar S. Müller M. F. (2021). Transcriptome-directed analysis for Mendelian disease diagnosis overcomes limitations of conventional genomic testing. J. Clin. Invest 131, e141500. 10.1172/JCI141500 33001864 PMC7773386

[B28] Pan J. Chia H. Kusnadi J. Li Z. Yu L. (2025). Renpenning syndrome related to a missense variant in polyglutamine-binding protein 1 (PQBP1): two pediatric cases from a Chinese family and literature review. Appl. Neuropsychol. Child., 1–9. 10.1080/21622965.2025.2457990 39932334

[B29] Pucheta-Martinez E. D'Amelio N. Lelli M. Martinez-Torrecuadrada J. L. Sudol M. Saladino G. (2016). Changes in the folding landscape of the WW domain provide a molecular mechanism for an inherited genetic syndrome. Sci. Rep. 6, 30293. 10.1038/srep30293 27456546 PMC4960638

[B30] Redin C. Gérard B. Lauer J. Herenger Y. Muller J. Quartier A. (2014). Efficient strategy for the molecular diagnosis of intellectual disability using targeted high-throughput sequencing. J. Med. Genet. 51, 724–736. 10.1136/jmedgenet-2014-102554 25167861 PMC4215287

[B31] Rejeb I. Ben Jemaa L. Abaied L. Kraoua L. Saillour Y. Maazoul F. (2011). A novel frame shift mutation in the PQBP1 gene identified in a Tunisian family with X-linked mental retardation. Eur. J. Med. Genet. 54, 241–246. 10.1016/j.ejmg.2011.01.010 21315190

[B32] Renpenning H. Gerrard J. W. Zaleski W. A. Tabata T. (1962). Familial sex-linked mental retardation. Can. Med. Assoc. J. 87, 954–956. 13981686 PMC1849750

[B33] Ropers H. H. (2008). Genetics of intellectual disability. Curr. Opin. Genet. Dev. 18, 241–250. 10.1016/j.gde.2008.07.008 18694825

[B34] Ropers H. H. (2010). Genetics of early onset cognitive impairment. Annu. Rev. Genomics Hum. Genet. 11, 161–187. 10.1146/annurev-genom-082509-141640 20822471

[B35] Ropers H. H. Hamel B. C. (2005). X-linked mental retardation. Nat. Rev. Genet. 6, 46–57. 10.1038/nrg1501 15630421

[B36] Růzicka E. Urgosík D. Jech R. Roth J. Vymazal J. Mecír P. (2005). Hemiparkinsonism and levodopa-induced dyskinesias after focal nigral lesion. Mov. Disord. 20, 759–762. 10.1002/mds.20453 15782419

[B37] Sheen V. L. Torres A. R. Du X. Barry B. Walsh C. A. Kimonis V. E. (2010). Mutation in PQBP1 is associated with periventricular heterotopia. Am. J. Med. Genet. A 152A, 2888–2890. 10.1002/ajmg.a.33507 20886605 PMC3548238

[B38] Stevenson R. E. Bennett C. W. Abidi F. Kleefstra T. Porteous M. Simensen R. J. (2005). Renpenning syndrome comes into focus. Am. J. Med. Genet. A 134, 415–421. 10.1002/ajmg.a.30664 15782410

[B39] Sudol M. McDonald C. B. Farooq A. (2012). Molecular insights into the WW domain of the golabi-ito-hall syndrome protein PQBP1. FEBS Lett. 586, 2795–2799. 10.1016/j.febslet.2012.03.041 22710169 PMC3413755

[B40] Tapia V. E. Nicolaescu E. McDonald C. B. Musi V. Oka T. Inayoshi Y. (2010). Y65C missense mutation in the WW domain of the golabi-ito-hall syndrome protein PQBP1 affects its binding activity and deregulates pre-IDNA splicing. J. Biol. Chem. 285, 19391–19401. 10.1074/jbc.M109.084525 20410308 PMC2885219

[B41] Wang Q. Moore M. J. Adelmant G. Marto J. A. Silver P. A. (2013). PQBP1, a factor linked to intellectual disability, affects alternative splicing associated with neurite outgrowth. Genes Dev. 27, 615–626. 10.1101/gad.212308.112 23512658 PMC3613609

