# Supplementary material for: Renpenning syndrome caused by the c.459_462delAGAG mutation in PQBP1: a case report and literature review
Source: Front Genet. 2026 Mar 30;17:1642438. doi: 10.3389/fgene.2026.1642438 (PMC13070661; doi:10.3389/fgene.2026.1642438)
Supplement: Supplementary file 1 [file DataSheet1.pdf]

The CARE Checklist

1. Title – The diagnosis or intervention of primary focus followed by the words "case report".

The title includes both the primary diagnosis and the phrase "case report."

2. Key Words – 2 to 5 key words that identify diagnoses or interventions in this case report (including "case report").

We included "case report" along with disease names and methods in the keywords: "Keywords: Renpenning syndrome, PQBP1, X-linked intellectual disability, whole exome sequencing, case report"

3. Abstract – (structured or unstructured)

a. Introduction – What is unique about this case and what does it add to the scientific literature?

This case reports the first Chinese patient with Renpenning syndrome caused by the specific PQBP1 c.459_462delAGAG variant. Its uniqueness lies in documenting the co-occurrence of the core phenotype with two rare features: anal atresia and autism spectrum disorder (ASD), thereby broadening the known clinical spectrum associated with PQBP1 mutations.

b. The patient's main concerns and important clinical findings.

The clinical focus centered on the child's severe global developmental delay, presenting with marked deficits in cognitive, language, and motor domains, as well as significant autistic features impacting social communication and adaptive functioning. Key clinical findings comprised a confirmed diagnosis of Renpenning syndrome supported by the identification of a pathogenic PQBP1 variant (c.459_462delAGAG), core manifestations including developmental delay, microcephaly, and short stature, a history of surgically corrected anal atresia, and a co-occurring diagnosis of ASD.

c. The primary diagnoses, interventions, and outcomes.

The primary diagnosis is Renpenning syndrome, resulting from a pathogenic PQBP1 variant, with comorbid ASD and a history of surgically corrected anal atresia. Interventions comprised the initial surgical repair at birth, followed by ongoing multidisciplinary rehabilitation and special education support. Follow-up to date indicates that, while global developmental delay and core autistic features persist with limited overall improvement, modest gains have been observed in the patient's language expression and social engagement.

d. Conclusion – What are one or more "take-away" lessons from this case report?

This case demonstrates that the PQBP1 c.459_462delAGAG variant can cause a clinical phenotype extending beyond the classic features of Renpenning syndrome to include anal atresia and ASD. It reinforces that whole exome sequencing (WES) is a crucial tool for the precise diagnosis of patients with unexplained intellectual disability, enabling definitive genetic counseling and guiding clinical management. A key lesson is that early genetic diagnosis in such cases can facilitate timely and tailored interventions.

4. Introduction – Briefly summarizes why this case is unique and may include medical literature references.

This case is unique as it represents, to our knowledge, the first report of Renpenning syndrome in a Chinese patient harboring the PQBP1 c.459_462delAGAG variant. A review of the existing literature on PQBP1-related disorders indicates that while intellectual disability, microcephaly, and short stature are well-established core features, the co-occurrence of anal atresia and a formal diagnosis of ASD with this specific variant has not been previously highlighted. Therefore, this report expands the recognized phenotypic spectrum associated with the c.459_462delAGAG mutation and underscores the importance of comprehensive phenotyping in genetic syndromes.

5. Patient Information

a. De-identified patient specific information.

As detailed in section 2.1 (Study subjects) of the manuscript, the proband is a 4-year-7-month-old Chinese male. His mother and both elder sisters are of normal intelligence, while his elder brother had intellectual disability and died prematurely. This family history pattern is consistent with X-linked inheritance. All personal identifiers have been removed to ensure patient privacy.

b. Primary concerns and symptoms of the patient.

As outlined in sections 2.1 Study subjects and 3.1 Clinical data analysis, the clinical presentation centered on the child's severe global developmental delay. This encompassed marked deficits in motor skills, cognition and language, along with significant impairments in social interaction. The core symptoms aligned with Renpenning syndrome, including developmental delay, microcephaly and short stature. The case was further characterized by a comorbid diagnosis of autism spectrum disorder and a history of anal atresia, which had been surgically corrected in infancy.

c. Medical, family, and psychosocial history including relevant genetic information.

Presented in section 2.1 Study subjects - see main manuscript:

“His able-bodied, non-consanguineous parents had four children: two boys and two girls. His two elder sisters were of normal intelligence, while his elder brother suffered from intellectual disability and died prematurely”. No significant psychosocial history was reported.

d. Relevant past interventions and their outcomes.

Past interventions primarily consisted of parent-led, home-based educational support and behavioral management. These efforts, however, have not led to significant symptomatic improvement; the child continues to exhibit marked global developmental delays and demonstrates limited engagement in peer interactions.

6. Clinical Findings – Describe significant physical examination (PE) and important clinical findings.

As described in section 3.1 (Clinical data analysis) of the main manuscript, “Objective measurements revealed microcephaly (occipitofrontal circumference 46.5 cm, < −3

SD), short stature (height 99.8 cm, < −2 SD), and lean body (weight 13 kg, < −3 SD). The special examination found that the child could only speak short sentences, had poor initiative social ability, less eye contact, liked to play alone, lacked joint attention, and could not participate in group games."

7. Timeline – Historical and current information from this episode of care organized as a timeline (figure or table).

NA.

8. Diagnostic Assessment

a. Diagnostic methods (PE, laboratory testing, imaging, surveys).

As detailed in the main manuscript (sections 3.1 to 3.5), the diagnostic evaluation involved a comprehensive approach, including physical and neurological examinations, collection of multi-center clinical data, and standardized developmental assessments. Laboratory investigations encompassed relevant blood tests to exclude metabolic and other systemic disorders, followed by genetic analysis via whole-exome sequencing with Sanger confirmation. Imaging studies included brain MRI/CT and urinary tract ultrasound, supplemented by electroencephalogram (EEG) and evaluations of vision and hearing.

b. Diagnostic challenges.

NA.

c. **Diagnosis (including other diagnoses considered).**

WES identified a hemizygous frameshift mutation in the PQBP1 gene NM_001032382.2:c.459_462delAGAG (p.Arg153fs) (VCV000010980.79). Sanger sequencing confirmed this variant was maternally inherited, consistent with X-linked recessive transmission, and it was classified as pathogenic according to ACMG guidelines. Therefore, the patient's condition is definitively diagnosed as Renpenning syndrome, caused by a pathogenic loss-of-function mutation in the PQBP1 gene.

d. Prognostic characteristics when applicable.

NA.

9. Therapeutic Intervention

a. Types of therapeutic intervention (pharmacologic, surgical, preventive).

As described in section 3.1 (Clinical data analysis) of the main manuscript, the patient has been receiving regular non-pharmacologic rehabilitative interventions, including weekly speech therapy and enrollment in a special education program. No surgical or pharmacologic treatments have been administered for the underlying genetic condition. To date, these supportive measures have contributed to modest gains in language ability and increased social responsiveness, such as more frequent use of facial expressions.

b. Administration of therapeutic intervention (dosage, strength, duration).

NA.

c. Changes in therapeutic interventions with explanations.

NA.

10. Follow-up and Outcomes

a. **Clinician- and patient-assessed outcomes if available.**

Outcome assessment relied on clinician evaluation through observation and developmental scales, as the patient's condition precluded self-reporting. Clinicians observed limited overall improvement, with persistent global delay and autistic features. Modest gains were noted in language and social interaction. Patient-reported outcomes were not available due to the patient's age and neurodevelopmental status.

b. Important follow-up diagnostic and other test results.

NA.

c. Intervention adherence and tolerability. (How was this assessed?)

NA.

d. Adverse and unanticipated events.

NA.

11. Discussion

a. Strengths and limitations in your approach to this case.

The key strengths of this case include the use of comprehensive genetic testing (WES) for definitive molecular diagnosis, followed by familial segregation analysis, which robustly confirmed the X-linked recessive inheritance pattern of the pathogenic PQBP1 variant. A systematic review of the literature further contextualized the genotype-phenotype findings. A primary limitation is the report of a single proband, which restricts broader generalization of the observed expanded phenotype. Additionally, the young age of the patient and relatively short follow-up period limit the assessment of long-term developmental trajectories and outcomes.

b. Discussion of the relevant medical literature.

The literature establishes that pathogenic variants in the PQBP1 gene cause Renpenning syndrome, with the core phenotype of intellectual disability, microcephaly, and short stature. The specific variant c.459_462delAGAG has been previously reported in association with this classic presentation. Our case aligns with these reports by confirming this variant in a patient exhibiting the core syndrome features. Furthermore, it extends the recognized phenotypic spectrum by documenting the co-occurrence of two rare features, specifically anal atresia and a formal diagnosis of autism spectrum disorder, in a patient harboring this variant, a combination not emphasized in prior publications. This observation suggests a broader clinical variability than previously described and underscores the importance of detailed phenotyping in genetic syndromes.

c. The rationale for your conclusions.

Our conclusions are based on the integration of definitive genetic evidence, consistent clinical findings, and a review of existing literature. The identification of a pathogenic PQBP1 variant (c.459_462delAGAG) via validated molecular methods provides the etiological basis for the diagnosis of Renpenning syndrome. The presence of anal atresia and ASD alongside the core features, while not previously highlighted for this variant, is supported by the patient's clinical data. The familial segregation pattern corroborates the X-linked recessive inheritance. Therefore, we conclude that this case expands the recognized clinical spectrum

associated with this specific PQBP1 mutation.

d. The primary "take-away" lessons from this case report (without references) in a one paragraph conclusion.

This case highlights the critical importance of comprehensive genetic testing, such as whole-exome sequencing, for patients presenting with unexplained intellectual disability and global developmental delay. This is especially relevant for individuals with a family history suggestive of X-linked inheritance, as a precise molecular diagnosis is fundamental to guiding clinical management and informing genetic counseling. Furthermore, it illustrates that within a defined genetic syndrome, individual variants can be associated with a broader clinical spectrum than previously recognized, underscoring the need for detailed phenotyping to fully elucidate genotype-phenotype correlations.

12. Patient Perspective– The patient should share their perspective on the treatment(s) they received.

Given the patient's young age and cognitive limitations related to his condition, obtaining a systematic personal account of his treatment experience was not feasible.

13. Informed Consent – The patient should give informed consent. (If not, explain)

The patient's legal representative provided written informed consent specifically for publication of this case report.
